# Instruction at the United Borough Hospitals in the Eighteenth Century

**Published:** 1903-07-25

**Authors:** 


### INSTRUCTION AT THE UNITED BOROUGH HOSPITALS IN THE EIGHTEENTH CENTURY.

MR. GEORGE PEACHEY has lately issued a very interesting account of William Savory, surgeon of Brightwalton in Berkshire. He says that in "1788, Oct. 3rd, I went to attend at St. Thomas's and Guy's Hospitals (the United Borough Hospitals) in London, and on Monday evening I went to Cline in St. Mary Axe, who is first surgeon and lecturer on anatomy at St. Thomas's Hospital. I paid him seven guineas for attending as a perpetual pupil to lectures on anatomy and surgery and five guineas for dissections. Mr. Cline's lecture this day was the fourth and on the absorbent vessels. I paid 2s. 6d. for a ticket on my entrance into the theatre. There are eighteen wards in St. Thomas's Hospital, twelve men's wards and six women's wards, including three wards called 'foul' wards for patients with the venereal disease. Cline gave seventy lectures on anatomy, the seventy-first dealing with the history of anatomy. These lectures were read again for the second course, which began in the January of each year. On April 22nd, 1789, began the lectures on surgery, which is always immediately after the spring course. In all there were twenty-one lectures on surgery, and after these Dr. Louder reads six or seven lectures on the gravid uterus at the theatre of St. Thomas's Hospital, and begins at half-past seven o'clock in the morning. The surgeon goes round the wards every day at eleven o'clock, every day by turns. Mr. Cline Mondays, Chandler Tuesdays, and Birch Wednesdays. Thursday is the day for taking in patients which is by turns, and on Saturday all go round, both physicians and surgeons. This Mr. Cline is an excellent surgeon, and is very near-sighted. In the large theatre at St. Thomas's, where Cline's lectures are given, is a table in the middle and seats running round it one above another: opposite is a little room containing an immense number of anatomical preparations, which are shown every day at the time of lecture, relating to what the subject is upon and on the other side of the staircase is the dissecting-room. October 15th, paid Dr. Louder five guineas to attend two courses of his lectures on midwifery. I went to his lecture every morning at half past seven o'clock till nine. His theatre is at his own house in St. Saviour's churchyard, near St. Thomas's and Guy's Hospitals, and on the other side of St. Saviour's Church is his labour-house. October 21st, began dissecting, and 29th came from my sister's in Whitechapel to a lodging-house in Joiner Street at number 32 with one Mrs. Meakin. I paid 2s. per week. The reason of my coming from my sister's being that it was so inconvenient in attending labours and accidents. The price for an adult subject is £2 2s., fœtus 7s. 6d., extremity 7s. 6d., head 7s. 6d., injecting a head 3s., injecting a placenta 2s. The first week I was at the hospital my head was greatly affected by going into the 'foul wards' and dissecting-room, but afterwards it did not hurt me. The most frequent operations performed were amputations and hernias. There was a lad about 14 years old, who had his leg amputated by Mr. Birch, when he astonished us all by being so cheerful during the time of the operation, and did not express any pain till tying the blood-vessels and then but little. Dr. Louder, the man midwife in St. Saviour's Churchyard, will not take a pupil for one course only, except he has attended somewhere else before. Two or three times a week there were labours at the lying-in house: we were all called and used to take them by turns. The doctor had a figure in the form of a woman and a little leather child to experience difficult labours and præternatural cases. After Cline's first course of lectures was ended I came into the country. I carried down with me two hearts injected, two arms and a fœtal skeleton. February 6, 1789, gave Dr. Saunders three guineas for attending one course of his lectures. He is an excellent lecturer and fine orator. During my time in London I was never at leisure, for at half past seven o'clock in the morning I went to Dr. Louder's lectures and stayed till nine. Then to breakfast, at ten to Dr. Saunder's lectures till eleven o'clock, then to the hospital till Cline's lecture, which was from one o'clock to three. By this time I used to get a good appetite for my dinner. The remainder of my time was taken up in writing lectures, clinical cases, attending labours and accidents, and in dissecting.

